# Time trends in psychosomatic symptoms among Hungarian youth using repeated cross sectional HBSC data from 2002 to 2022

**DOI:** 10.1038/s41598-026-38472-0

**Published:** 2026-02-06

**Authors:** Mária Klein, Dóra Várnai, Ágnes Németh, Zsolt Horváth, Gyöngyi Kökönyei

**Affiliations:** 1 Doctoral School of Psychology, ELTE Eötvös Loránd University, Budapest, Hungary; 2 Institute of Psychology, ELTE Eötvös Loránd University, Izabella Street 46, Budapest, 1064 Hungary; 3 Heim Pal National Institute of Pediatrics, Budapest, Hungary; 4 NAP3.0-SE Neuropsychopharmacology Research Group, National Brain Research Program, Semmelweis University, Budapest, Hungary; 5 Department of Pharmacodynamics, Faculty of Pharmaceutical Sciences, Semmelweis University, Budapest, Hungary; 6 Centre of Excellence in Responsible Gaming, University of Gibraltar, Gibraltar, Gibraltar

**Keywords:** Diseases, Health care, Medical research, Psychology, Psychology, Signs and symptoms

## Abstract

Psychosomatic symptoms have become increasingly prevalent among adolescents; however, trends covering a longer period remain underexplored in Hungary. This study examines changes in psychosomatic symptoms among school-aged children using nationally representative data from the Hungarian Health Behaviour in School-aged Children (HBSC) survey, collected in 2002, 2006, 2010, 2014, 2018, and 2022 (N = 37,930; M_age_ = 14.88 years, SD_age_ = 2.36 years; boys = 49.3%). Descriptive statistics, logistic regression analyses, and ANCOVAs were conducted to assess trends from 2002 to 2022. Girls and older adolescents reported more symptoms overall, with 2022 showing the highest prevalence across all symptom categories in both genders. The proportion of adolescents reporting multiple health complaints (MHC) (excluding fatigue) became considerably more common between 2010 and 2022, increasing from 40.6 to 65.6% among girls, and from 30.2 to 42% among boys. Fatigue emerged as the most reported frequent symptom across all survey waves among both genders, peaking in 2022 at 47.5% among boys and 67.6% among girls. The findings point to a trajectory—particularly in recent years—highlighting the urgent need for targeted interventions and policy actions to support adolescent health.

**Supplementary Information:**

The online version contains supplementary material available at 10.1038/s41598-026-38472-0.

## Introduction

Although morbidity and mortality rates^1^ are important indicators of adolescent health, subjective health indicators, such as self-reported (subjective) health complaints, are also play a crucial role in characterizing the health conditions of this population^2^. Subjective health complaints, also referred to as psychosomatic symptoms, are frequently discussed in scientific literature^3,4^. Psychosomatic symptoms refer to subjective physical complaints—such as headaches or back pain—as well as psychological complaints, including feelings of low mood or irritability^5^. It is important to note that the terms “psychological” and “somatic” cannot be interpreted as the causes of the symptoms^6^. Therefore, subjective health complaints are suggested as a neutral descriptive term that highlights the personal experience of how these symptoms affect an individual’s life. In this paper, we will prefer to use the term psychosomatic symptoms. Studies have shown that psychosomatic complaints are relatively common among young people^7–9^. These symptoms do not necessarily impact the daily functioning of youth negatively^10^; however, they may have a relationship with negative health outcomes^11^, such as impaired life satisfaction of students^12^, school-related distress^5^, and increased screen time usage^13^. Furthermore, persistent symptoms reported in childhood can predict serious health issues, manifesting as symptoms of depression or anxiety in later stages of life^14^. Additionally, psychosomatic symptoms, such as headaches and sleeping difficulties, may be associated with the emergence of somatic complaints in adulthood^15^. Analysing trend patterns of somatic and psychological symptoms in school-aged children provides a unique opportunity to gain insight into youth’s health over time. Tracking these trends may also facilitate the identification of broader social, cultural, and economic influences on symptom prevalence. The repeated cross-sectional Health Behaviour in School-aged Children (HBSC) data^16^, collected every four years, suggest that trend patterns in symptoms vary considerably across countries^17^. Therefore, analysing trends at the national level is important, as it may also enable the identification of broader social, cultural, and economic influences on symptom prevalence in a given country. The objective of this study is to examine temporal changes in psychosomatic symptoms among Hungarian pupils from 2002 to 2022. The Hungarian HBSC data set merits particular attention and analysis because, unlike international HBSC reports which include only 11-, 13-, and 15-year-olds, the Hungarian HBSC studies also included 17-year-olds. In this age group, issues related to learning and the future life course emerge strongly, possibly influencing overall health. Furthermore, in addition to the eight common symptoms covered by international HBSC analyses (see below), Hungarian surveys also assess the prevalence of frequent fatigue.

Central and Eastern European (CEE) countries, including Hungary, have followed distinct developmental trajectories compared to Western Europe, shaped by historical, socio-cultural, and geopolitical factors^18^. Macro-level indicators, such as Gross National Income (GNI) per capita, might influence the development of psychosomatic symptoms, although the underlying mechanisms are likely complex and multifactorial^17^. While Hungary is currently classified as a high-income country^19^, it continues to lag behind Western European nations in terms of income and broader economic development. Despite progress in poverty reduction, significant regional and demographic disparities remain^20^, especially in rural areas. These persistent inequalities, common across CEE countries, underscore the need to monitor health outcomes over time. Notably, these disparities are also reflected in broader population health indicators. In 2023, life expectancy at birth in Hungary was 76.7 years—considerably below the EU average of 81.4 years, and also lower than in countries such as Poland (78.4 years), Slovakia (78.2 years), and Estonia (79.1 years)^21^. There is robust evidence that trajectories of health and life expectancy are strongly influenced by exposures and health status in early life^22^, which underscores the importance of monitoring adolescent health indicators. The absence of a national mental health strategy in Hungary^23^ further highlights the importance of such research. Although schools are legally required to provide comprehensive health education—including mental health—since 2021, only accredited non-governmental organizations (NGOs) are permitted to deliver these services. Meanwhile, several NGO-led initiatives aim to address adolescent mental health challenges; however, access to these services is often limited for young people. According to the findings of a recent representative survey of almost 10,000 young people aged 14–29 years from CEE and Baltic countries, Hungary had the highest proportion of youth reporting very low life satisfaction with 16% chose the lowest response categories (“very” or “mostly” dissatisfied)^24^. This aligns with the pattern that Hungarian youth report comparatively poorer subjective well-being indicators than young people in neighbouring countries. Hungary’s experience may thus offer important insights for other CEE nations seeking to understand how socio-economic disparities affect adolescent health, and to inform intervention strategies in similar regional contexts.

Several empirical studies have investigated the trend of psychosomatic symptoms based on data from international HBSC studies between various time ranges. Most of these studies indicate an increase in self-reported psychosomatic symptoms among school-aged children in European countries generally, including Hungary between 2002 and 2018^25^. County specific studies, for example in Sweden from 1985 to 2005^26^, in France from 2002 to 2014^27^, and in Scotland from 1998 to 2018^28^ further underline this trend. A systematic review and meta-analysis, covering the period from 1983 to 2013, supports the increasing trend of psychosomatic symptoms as well^29^. The results of an Austrian study^30^ showed quite the opposite: a decreasing trend was observable when analysing the trend of psychosomatic symptoms between 1994 and 2006. Ottová-Jordan and colleagues^31^ expanded their analysis to include countries in North America, Europe, and Israel. Their results revealed varying trends in multiple health complaints (MHC), defined as the presence of two or more health complaints occurring at least once a week. They found that the trend remained stable in Hungary between 1994 and 2010. In contrast, the HBSC international report from 2022, assessing trends from 2014 through 2018 to 2022, shows a worsening trend in MHC for both genders^32^. The pattern of MHC is particularly noteworthy, as it highlights a significant level of complaints.

The results of papers aiming to explore the time trend of psychosomatic symptoms based on data other than the HBSC data showed similar findings to the HBSC-based papers. Most of these studies demonstrated an increase in psychosomatic symptoms, e.g., among Swedish children from 1984 to 2011^33^. Some papers indicated a stable trend^34^, while others showed a decreasing trend over time, e.g. among adolescents in the United Kingdom between 1999 and 2004^35^. It is important to note, regardless of whether the studies are HBSC-based or non-HBSC based, gender differences are observable in almost every paper; girls report more psychosomatic symptoms than boys^17,36,37^. In addition to gender, age plays a significant role in the development of symptoms, with older adolescents experiencing more psychosomatic symptoms^38^. Moreover, the COVID-19 pandemic has further exacerbated this trend, as numerous studies have documented a worsening of adolescent well-being during this period^39–41^. The temporal rise of psychosomatic symptoms is consistent with evidence showing increases in adolescent internalising problems more generally. Recent meta-analytic evidence indicates that globally the point prevalence of elevated depressive symptoms among adolescents increased from approximately 24% (2001–2010) to around 37% (2011–2020), with girls reporting higher rates than boys^42^.

Besides the differences in age and gender regarding symptoms, there could also be differences in the prevalence of the subjective health complaints. Furthermore, the general symptom-based approach often masks the complexities of individual complaints. Fatigue is a particularly important and frequently reported symptom among adolescents, often attributed to puberty-related hormonal changes, psychological challenges, and increased demands in educational and social contexts^43^. Nevertheless, the high prevalence of fatigue does not stop in adolescents; it extends into adulthood as well. For example, in a previous study^44^, 65% of individuals aged 15 to 84 years reported tiredness, or in other words, fatigue. The Hungarian HBSC incorporates all mandatory questions from the national test battery and expands upon them by including an optional question about “fatigue”^45^. Without any doubt, it is important to have a more nuanced understanding of how individual symptoms fluctuate over time, such as identifying which symptoms are most or least prevalent, or which symptoms show the greatest increase over time. The individual-symptom approach could foster better-tailored treatments, and a more comprehensive understanding of the subjective health.

Taken together, the aim of this study is to gain a comprehensive picture of the subjective health condition of Hungarian pupils by examining the temporal changes in psychosomatic symptoms among Hungarian pupils between 2002 and 2022 based on the Hungarian HBSC data. In this study we specifically aim (1) to describe the temporal pattern of the individual symptoms including those observed during the COVID-19 period; and (2) analyse the changes in MHC over time, and (3) investigate the changes in the total score of psychosomatic symptoms over time. To facilitate comparison of our results with previous findings from other HBSC studies, we present results both excluding and including fatigue. The results excluding fatigue will be referred to as the "8-symptom version" e.g., 8-symptom version of the HBSC-Symptom Checklist (HBSC-SCL)^46^, while results including fatigue will be marked as the "9-symptom version" (e.g., 9-symptom version of the HBSC-SCL).

## Methods

### Study population and procedure

The present study utilised Hungarian datasets from the international HBSC studies collected in the years 2001/2002, 2005/2006, 2009/2010, 2013/2014, 2017/2018 and 2021/2022. To simplify, we will refer to the survey year 2001/2002 as 2002, 2005/2006 as 2006, 2009/2010 as 2010, 2013/2014 as 2014, 2017/2018 as 2018, and 2021/2022 as 2022. HBSC is a WHO collaborative cross-national research study that is conducted every four years across Europe and North America. Its primary aim is to provide comprehensive insights into the health-related lifestyle of school-aged adolescents using self-reported questionnaires. The Hungarian HBSC study adhered to both HBSC guidelines and International Protocols^16,47^. The final dataset included results from all six survey years (2002: n = 5,986; 2006: n = 5,450; 2010: n = 8,096; 2014: n = 6,153; 2018: n = 6003; and 2022: n = 6,291), totalling 37,979 participants. The samples were always representative in every survey year, ensuring reliable population estimates across the study period. After excluding 35 students who did not respond to the gender question and 14 whose ages fell significantly outside the typical range for school-age students, our final sample consisted of 37,930 participants. This final sample (N = 37,930) provided representative data from 5th-, 7th-, 9th-, and 11th-grade students based on the latest statistics of Hungarian public education. The age range of the adolescents was 9 to 21 years (*M* = 14.88, *SD* = 2.36), and the gender distribution was 49.3% boys.

The principals of the selected schools were invited by letter to participate in the HBSC research. Upon their agreement, the students’ parents were informed in writing about the study, and parental consent was sought for their child’s participation. Students completed the internationally standardized questionnaire on a voluntary basis, with parental passive consent being required. The survey was administrated in school classrooms, and it took approximately 45 min to complete. In 2022, the participants completed the questionnaire online, while in the other survey years, paper-based questionnaires were used. Previous studies have shown that the mode of administration had no significant effect^48^ or only a minor significant effect in reporting exercise^49^ when comparing paper-based and electronically administered questionnaires among adolescents, suggesting comparable results from both methods.

To ensure full anonymity, only a trained interviewer was present during the completion of the questionnaire. After completing the survey, pupils returned their questionnaires in sealed envelopes, which were collected by the interviewer. The Hungarian HBSC surveys were conducted with the approval of the Scientific and Research Ethics Committee of the Health Scientific Council.

### Measures

#### Psychosomatic health complaints

Subjective health complaints were assessed using the international HBSC-SCL^46^. This non-clinical, self-report measure consists of eight psychosomatic items: headache, stomach-ache, backache, feeling low, irritability (or bad mood), feeling nervous, sleeping difficulties, and dizziness. For the present analysis, the Hungarian version of the HBSC-SCL was utilised, which includes fatigue as a ninth psychosomatic complaint^45^. Respondents were asked to indicate how often they had experienced one or more of these health complaints over the past six months, using the following response options: (1) ‘About every day’, (2) ‘More than once a week’, (3) ‘About every week’, (4) ‘About every month’, (5) ‘Rarely or never’. For the statistical analyses, we reverse-scaled the raw values of the scale, with higher values indicating more frequent symptoms. Frequent individual symptoms were created by dichotomising the individual symptoms, assigning a value of 1 to symptoms occurring with ‘More than once a week’ or ‘About every day’ frequency, and a value of 0 to all other response options. This indicates that pupils who reported experiencing a symptom frequently have it at least more than once a week.

MHC were defined following previous recommendations^12^; the presence of MHC was indicated by reporting at least two of the eight symptoms occurring more than once a week. The total score of the symptoms was calculated by summing up the reversed point scores of the 8-symptom (without fatigue) and the 9symptomversions of the HBSC-SCL. The total scores ranged from 8 to 40 for the 8-symptom version, and 9 to 45 for the 9-symptom version of the HBSL-SCL, where higher scores indicate more frequent and severe health complaints. A previous paper^50^ has demonstrated that the 8-item version of HBSC-SCL exhibits good internal reliability, with Cronbach *α* = 0.88. In our research, both versions of the HBSC-SCL were found to be highly reliable (8-symptom-version *α* = 0.853, 9-symptom-version *α* = 0.866).

#### Demographic variables

Gender was determined by asking respondents if they identified as male or female. Age was calculated using the respondents’ date of birth and the date of survey completion.

#### Perceived school pressure

Perceived pressure from schoolwork was assessed with a single item asking pupils how pressuring they find their school tasks, rated on a 4-point scale (‘1 = not at all’, ‘4 = very much’). For the additional descriptive analysis, high school pressure was defined as responses scored as 4.

#### Data analytic plan

All the analyses were carried out using IBM SPSS Statistics for Windows, version 29.0 (IBM Corp., Armonk, New York, United States). Diagrams were generated using R software version 4.4.1 R Core Team^51^.

Due to declines, school absences, and data cleaning procedures, any violations of representativeness were corrected by applying post-stratification weights by geographical location, type of habitation (e.g., capital city, city, and village), type of school (e.g., primary school, vocational school, and high school), and operator of school (e.g., municipal council and church) in each survey year. Based on the statistics of the educational institutions in the 2002 data collection year, there was no possibility of determining the statistical weights retrospectively, so we applied a weight of 1 for this year. We conducted the statistical tests both with and without weighting, and the reported results reflect the weighted version. Due to our substantial dataset (*N* = 37,930), we performed parametric tests. The missing data for the individual symptoms were minimal (ranging from 56 to 108 in 2002, from 91 to 134 in 2006, from 93 to 140 in 2010, from 130 to 176 in 2014, from 78 to 127 in 2018, and from 162 to 190 in 2022). The missing data were not imputed.

Firstly, we conducted descriptive statistics to summarize the characteristics of our sample. Secondly, to assess the temporal changes in individual frequent symptoms over time, the chi-square test was performed to investigate the impact of survey year on the occurrence of each individual frequent symptom. To report the odds of experiencing each symptom frequently, binary logistic regression was conducted with the individual frequent symptoms as the outcome, while using survey year (reference = 2002) and gender (reference = boys) as predictors, controlling for age. The predictor variables were entered into our models using the Enter method. The 95% confidence intervals for odds ratios were also presented. Model assumptions for these logistic regression models were checked via inspection of multicollinearity indices and residual diagnostics.

Thirdly, chi-square tests were performed to determine the impact of survey year on the presence of the 8- and 9-symptom versions of MHC. We also performed descriptive comparisons of grade-level differences in the 8-symptom version of MHC. In addition, perceived school pressure was examined solely descriptively to explore whether its pattern aligned with the temporal trends; this variable was not analysed as an outcome or predictor in any statistical model. Subsequently, to assess the odds of experiencing both versions of MHC, binary logistic regression was conducted, with the 8- and 9-symptom versions of MHC defined as the outcome variable, while survey year (reference = 2002) and gender (reference = boys) as predictor variables, controlling for age. The Enter method was used the enter the predictor variables, and the 95% confidence intervals for odds ratios were also presented. The analytic plan and model specifications for these models were pre-defined prior to running the analyses to avoid data-driven model selection.

Lastly, one-way analyses of covariance (ANCOVAs) were performed to assess the impact of survey year on the total scores of the 8- and 9-symptom versions of the HBSC-SCL, with age serving as a covariate. Survey year was used as a polynomial variable to identify the trend patterns of the symptoms. Model diagnostics indicated that ANCOVA assumptions were met.

As the temporal pattern of psychosomatic symptoms reported by boys and girls differed significantly across individual symptoms, total scores, and MHC, the results are presented separately for each gender. In reporting the results, we prioritised presenting both odds ratios with 95% confidence intervals and absolute percentage point changes to enhance interpretability of effect sizes.

#### Merging the six HBSC datasets

Before conducting statistical analyses, we merged the six HBSC datasets together based on the variables of interest. The original datasets were stored as SPSS files, with each questionnaire completed under a unique ID number. For the purposes of this study, we included only the nine psychosomatic symptoms that were consistently present across all six data collection years. As a result, we excluded the symptom options ‘I was scared’, ‘I woke up several times’, and ‘I felt nauseous and vomited’ from the 2002 dataset.

## Results

### The prevalence of individual frequent symptoms

Table 1 presents the prevalence of individual frequent symptoms by gender and survey year. The data show a generally increasing trend in psychosomatic symptoms among both boys and girls from 2002 to 2022. Girls reported higher prevalence rates across all symptoms and survey years compared to boys. Fatigue was the most prevalent symptom in every survey year for both boys and girls, while dizziness consistently had the lowest prevalence in both genders across all survey years. These results suggest a general intensification of symptom burden across two decades, with especially steep increases in symptoms that reflect chronic stress and physiological arousal (fatigue, sleeping difficulties). The pattern indicates that psychosomatic symptoms are increasingly embedded in the everyday experience of Hungarian adolescents.

**Table 1:** Prevalence of frequent psychosomatic symptoms by survey year and gender.

Symptom	Survey year	Boys		Girls	
		%	N	%	N
Frequent headache	2002	9.3	251	25.9	828
	2006	14.6	402	30.0	782
	2010	15.0	616	27.3	1,062
	2014	12.5	360	28.5	893
	2018	14.1	408	29.3	884
	2022	18.2	539	37.2	1,179
Frequent stomach-ache	2002	6.5	175	16.7	533
	2006	8.6	236	20.1	524
	2010	10.1	415	18.1	704
	2014	8.6	248	18.7	586
	2018	10.0	288	21.4	646
	2022	13.5	400	30.2	952
Frequent backache	2002	9.6	255	14.7	466
	2006	11.5	312	16.9	437
	2010	12.9	525	17.2	667
	2014	12.8	367	19.7	613
	2018	13.7	395	19.0	570
	2022	18.6	549	29.9	943
Frequent feeling low	2002	16.3	436	27.9	888
	2006	19.3	530	28.0	727
	2010	16.8	688	22.8	888
	2014	18.8	538	29.0	903
	2018	20.2	583	33.0	991
	2022	28.8	851	49.0	1,546
Frequent irritability	2002	16.0	430	21.8	696
	2006	16.8	460	23.1	598
	2010	17.2	704	20.6	798
	2014	14.1	401	25.3	788
	2018	17.5	506	27.1	815
	2022	24.2	715	44.2	1,396
Frequent feeling nervous	2002	26.1	703	33.6	1,071
	2006	27.4	754	35.1	907
	2010	26.2	1,072	30.1	1,166
	2014	22.4	642	32.7	1,018
	2018	27.0	780	38.1	1,145
	2022	37.8	1,114	55.5	1,749
Frequent sleeping difficulties	2002	11.5	307	17.7	566
	2006	13.1	359	18.4	475
	2010	15.7	645	19.0	736
	2014	14.8	422	23.7	738
	2018	18.3	527	28.5	855
	2022	25.3	745	39.1	1,236
Frequent dizziness	2002	5.3	141	11.6	367
	2006	6.9	188	11.7	302
	2010	8.4	342	11.9	461
	2014	7.1	201	11.2	347
	2018	7.5	216	14.4	431
	2022	10.3	303	23.8	753
Frequent fatigue	2002	29.0	777	39.3	1,247
	2006	35.3	969	42.8	1,114
	2010	37.0	1,515	41.5	1,607
	2014	38.0	1,090	48.5	1,513
	2018	41.8	1,205	51.8	1,554
	2022	47.5	1,403	67.6	2,136

A symptom was considered frequent when it was reported as occurring at least ‘More than once a week’ over the past six months.

Among the psychosomatic symptoms, associations between irritability, feeling low and feeling nervous were the strongest (Pearson’s *r* around 0.600, *p* < 0.01), while the association between backache and sleeping difficulties was the weakest (Pearson’s *r* = 0.314, *p* < 0.01). Age had a significant, but weak correlation with the individual symptoms (Pearson’s *r* ranged from 0.052 to 0.187, with p values *p* < 0.01). For the correlation matrix of the nine individual symptoms in the total sample, see Table S1 in the Supplement.

### Temporal changes in the individual frequent symptoms

**Fig. 1:**
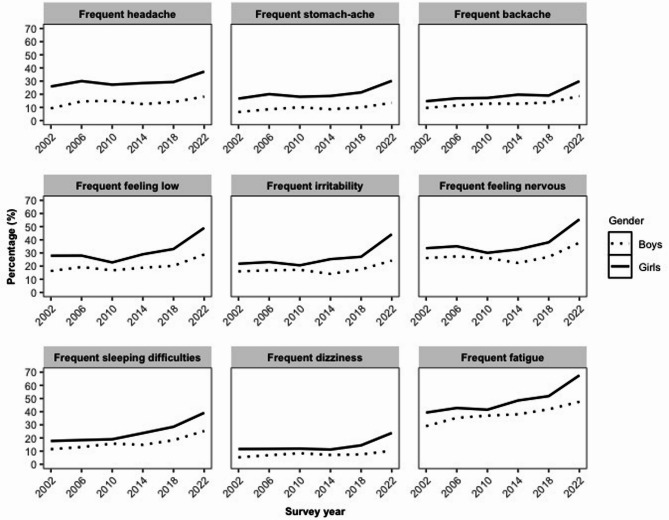
Changes in the prevalence of each frequent psychosomatic symptom over time by gender. *Note.* A symptom was considered frequent when it was reported as occurring at least ‘More than once a week’ over the past six months.

According to the results of the chi-square tests, there was a significant association between all survey years and each individual frequent symptom in both genders. For the detailed results see Table S2 in the Supplement. The crosstabulations showed that girls experienced a higher occurrence of each frequent symptom in every year than boys. Each frequent symptom exhibited its highest prevalence in 2022 for both genders. It is also noteworthy that the proportion of students reporting frequent symptoms of pain, dizziness, and sleep difficulties increased approximately twofold by 2022 for both genders compared to the 2002 data (Table 1). Nevertheless, concomitantly, the proportion of students reporting frequent irritability, low mood, and nervousness has also been found to be elevated in 2022, particularly among girls. The frequent fatigue symptom is particularly notable: it showed the highest prevalence among all symptoms for both genders across all survey years, peaking in 2022 at 47.5% for boys and 67.6% for girls. Frequent dizziness was the least common symptom across all survey years in both genders, reaching its lowest rates in 2002at 5.3% for boys and in 2014 at 11.2% for girls. For the changes in the prevalence of school-aged children experiencing each frequent symptom, see Table 1. A gender-specific comparison of temporal changes in the percentage of pupils reporting each frequent symptom is illustrated in Fig. 1.

### Binary logistic regression on the individual frequent symptoms

Girls were more likely to report each of the nine frequent psychosomatic symptoms compared to boys, with odds ratios ranging from 1.46 (for frequent feeling nervous) to 3.45 (for frequent headache). This indicates, for example, that the odds of a Hungarian school-aged girl experiencing a frequent headache were 245% higher than those of a boy. When comparing the effects of the survey year, the greatest differences were observed between 2002 and 2022, with odds ratios varying from 1.63 (for frequent irritability) to 2.61 for frequent sleeping difficulties. Among the symptoms, frequent fatigue and frequent headache stand out; each survey year showed a significant increase (*p* < 0.001) when compared to 2002. The effect of age was significant for every frequent symptom. The interaction between gender and survey year was significant for several outcomes. This indicates that the odds of reporting frequent psychosomatic symptoms varied by both gender and survey year. Specifically, girls in later survey years (2010, 2014, 2018, and 2022) had different odds ratios compared to boys in 2002, with significant interaction effects observed in multiple frequent symptom categories. Thus, the gender gap is not static but has widened over time, indicating that girls are disproportionately affected by the worsening symptom environment. This highlights that gender is not merely a background covariate, but an active driver shaping how symptom trends unfold. For detailed results, see Table S3 in the Supplement.

### Temporal changes in the 8-symptom version of MHC

**Fig. 2:**
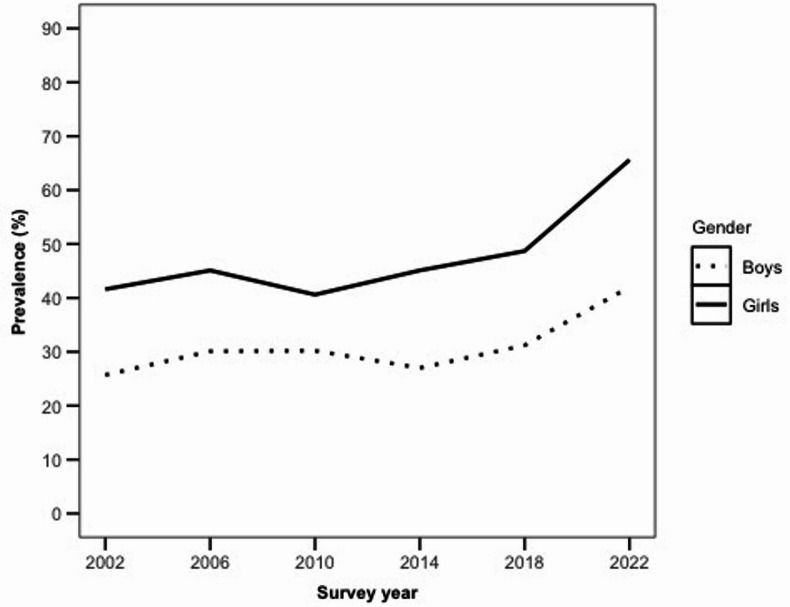
Changes in the prevalence of pupils reporting 8-symptom version of MHC over time, separated by gender (without fatigue). *Note*. MHC = multiple health complaints, excluding fatigue.

A chi-square test of independence was conducted to evaluate the relationship between survey year and the 8-symptom version of MHC. The results showed a significant association between these variables for both genders: Boys: *χ*2 (5, *N* = 18,375) = 228.08,* p* < 0.001; Girls: *χ*2 (5,* N* = 19,067) = 551.40, *p* < 0.001. The corresponding effect sizes were small-to-moderate (Cramér’s *V* = 0.11 for boys; Cramér’s *V* = 0.17 for girls). These results suggest that the prevalence of the 8-symptom version of the MHC has significantly increased over the survey years. The lowest prevalence of symptoms was measured in 2002 for boys (25.7%) and in 2010 for girls (41.6%), while the highest prevalence was observed in 2022 for both boys (42.0%) and girls (65.6%). In each survey year, a higher percentage of girls reported the 8-symptom version of MHC compared to boys (see Fig. 2). Table S4 in the Supplement displays the percentages of pupils reporting the 8-symptom version of MHC by survey year and gender.

When comparing the grades, the most prominent changes occurred in the upper grades. Among boys, 11th grade increased to nearly half reporting MHC in 2022 (49.0%). Among girls, the escalation in 9th and 11th grade is dramatic: 9th graders rose to 72.3% and 11th graders to 76.4% in 2022. For detailed comparisons, see Table S5–S6 and Fig. S1–S2 in the Supplement.

### Binary logistic regression on the 8-symptom version of MHC

The binary logistic regression analysis indicates that girls were significantly more likely than boys to report the 8-symptom version of MHC across all survey years. The odds ratios for experiencing MHC without fatigue were highest in 2022. Hungarian school-aged youth in 2022 were 131% more likely to experience the 8-symptom version of MHC compared to 2002. Age also had a significant effect on the likelihood of reporting the 8-symptom version of MHC; older pupils consistently showed higher risk than younger ones. The observed increase appears to reflect a broad elevation across multiple psychosomatic domains rather than a change in one single symptom. Thus, the increase represents a shift towards a more widespread and multi-domain symptom burden in the adolescent population. For detailed results, see Table 2.

**Table 2:** Results of binary logistic regression for the 8-symptom version of MHC (without fatigue).

Variables	OR [95% CI]
Gender (ref = Boys)	
Girls	2.07*** [1.95, 2.17]
Survey Year (ref = 2002)	
2006	1.18*** [1.09, 1.27]
2010	1.04 [0.97, 1.19]
2014	1.05 [0.98, 1.14]
2018	1.29*** [1.19, 1.40]
2022	2.31*** [2.14, 2.49]
Age	1.12*** [1.11, 1.13]

The reference categories are boys for gender and the year 2002 for the survey year. The variables were entered into the model using the Enter method. MHC = MHC, excluding fatigue. OR = Odds Ratio, CI = Confidence Interval.

*** p < 0.001.

### Additional analyses on the 9-symptom version of MHC

We conducted chi-square test of independence and binary logistic regression on the 9-symptom version of MHC, which includes fatigue as the ninth symptom in addition to the eight standard HBSC complaints. The results of both analyses on the 9-symptom version of MHC were consistent with the previous findings for the 8**-**symptom version of MHC. Chi-square test: Once again, the survey year showed a significant association with the occurrence of the 9-symptom version of the MHC: Boys:* χ*2 (5, *N* = 18,376) = 235.56, *p* < 0.001; Girls:* χ*2 (5, *N* = 19,066) = 528.09, *p* < 0.001. As expected, adding fatigue to the 8-symptom index increased the prevalence rates across all survey years, but the overall pattern remained unchanged, with the lowest prevalence observed in 2002 (32.3% for boys) and in 2010 (47.5% for girls) and the highest rates recorded in 2022 for both boys (50.0%) and girls (71.7%) (see Table S7 in the Supplement). The binary logistic regression results indicated that girls and older adolescent had significantly higher odds of experiencing the 9-symptom version of MHC. For detailed results, see Table S8 and Fig. S3 in the Supplement.

### Additional analyses on the total scores for the 8- and 9-symptom versions of the HBSC-SCL

In addition to the MHC approach, we performed further analysis using one-way ANCOVAs with the outcome variables from both the 8- and 9-symptom versions of HBSC-SCL, with age serving as a covariate. The results of the one-way ANCOVA tests are in line with the symptom patterns revealed by the chi-square test and binary logistic regression for both the 8- and 9-symptom versions of the MHC. As in previous analyses, girls reported higher levels of symptoms than boys in every survey year. For boys, the lowest values were observed in 2002 for both versions, whereas for girls the lowest values occurred in 2010 for the 8-symptom version and in 2002 for the 9-symptom version of the HBSC-SCL. The highest values were measured in 2022 for both genders, regardless of age. This consistency across analytical approaches reinforces the robustness of the observed trend: no matter whether symptoms are examined individually, as multiple complaints, or as overall scale scores, the pattern is the same—2022 is the peak, and girls are most affected. For detailed results, please refer to Supplement S4.

### Additional descriptive analyses on perceived school pressure

To contextualise the symptom trends, we also examined changes in perceived school pressure. The proportion of pupils reporting that school tasks felt very pressuring increased over time, rising from 6.8 to 9.5% among boys and from 5.6 to 14.1% among girls between 2002 and 2022. The steepest increase occurred between 2018 and 2022, particularly among girls. Full descriptive statistics and the corresponding figure are provided in Supplement Table S11 and Fig. S6.

## Discussion

Given the considerable variation in trend patterns in health indicators such as psychosomatic symptoms in the school-aged population across countries^17^, analysis at the national level is imperative. The present analyses were thus planned to examine cross-sectional trends in psychosomatic symptoms among Hungarian school-aged children over the past two decades. In summary, the findings indicate an overall worsening trend between 2002 and 2022, with a particularly pronounced increase among older adolescents and girls. When analysing symptoms that occur with high frequency, significant gender differences were observed across all symptoms. The most pronounced changes observed between 2002 and 2022, affecting both genders, were evident in frequent sleeping difficulties, dizziness, headache, backache, and stomach-ache. By 2022, the proportion of students reporting these symptoms increased twofold for both genders compared to the 2002 data. Moreover, a high proportion of youth reported experiencing frequent nervousness, irritability, feeling low, and fatigue in 2022. The interaction effects between gender and survey year reveal that, particularly in 2010 and 2020, girls had significantly higher odds of experiencing psychosomatic symptoms compared to boys in 2002. The pattern of frequent individual symptoms was reflected in the prevalence of MHC, as well as in the total scores for psychosomatic symptoms. The most substantial differences were observed between 2002 and 2022, with the highest symptom levels recorded in 2022, especially among girls and older adolescents, who were disproportionately affected. The exceptionally high proportion of school-aged children reporting both the 8- and the 9-symptom versions of MHC in 2022 is particularly concerning, as it suggests a substantial symptom burden at the population level and warrants public health attention, while not constituting a clinical diagnosis. In addition, the grade-specific analyses revealed that the steepest increases in the 8-symptom version of MHC occurred in the upper grades. This indicates that psychosomatic symptom burden intensifies toward the end of compulsory schooling, which may be consistent with increasing educational and developmental demands in late adolescence, although causal interpretations cannot be drawn from the present design. Changes in total symptom level scores reflect a continuous increasing trend from 2010 onward among girls, and from 2014 onward among boys. While the inclusion of fatigue has naturally increased the occurrence of MHC and elevated total symptom scores, it has not altered the underlying trend.

In line with previous studies, the increase in psychosomatic symptoms is more pronounced among girls than boys^52,53^, a difference attributed to various factors. For example, De Looze and colleagues^53^ argue that in recent years, the association between national-level gender inequality and adolescent mental health has shifted, becoming negative—potentially due to rising academic pressure. This trend is further compounded by the fact that girls are generally more vulnerable to interpersonal stress than boys, particularly in peer and family contexts^54^. For example, a withdrawal from friendship for girls maybe perceived as a threat to self-esteem, potentially elevating stress levels and thereby contributing to increased symptoms of depression and anxiety. Previous longitudinal study^55^ has identified body shame—a feeling ashamed of one’s body—as a factor that may exacerbate depressive symptoms among young girls. Furthermore, girls are often socialised to express emotional distress more openly than boys, potentially leading to a higher reporting rate of such symptoms^56^. Besides gender differences, age also showed a significant association with psychosomatic symptoms. The Hungarian HBSC surveys analysed here also include older adolescents (11th grade, *M*_age_ = 17.77 years, *SD*_age_ = 0.73 years), and a body of research indicates that older adolescent report more psychosomatic symptoms than younger ones^17,57^.

Upon examining changes in individual symptoms, we observed that while most symptoms fluctuated from 2002 to 2018, but from 2018 onwards, each frequent symptom demonstrated an upward trend, peaking in 2022. This increase aligns with numerous papers reporting deteriorating among youth during the COVID-19 pandemic, with heightened levels of anxiety, depression, and suicidal ideation^58,59^. Fatigue emerged as the most prevalent symptom over the twenty years for both genders, with 47.5% of boys and 67.6% of girls experienced fatigue at least once a week in 2022. However, this result is not surprising, giving that fatigue is common among adolescents due to the biological and psychological transitions of puberty^43^. Additionally, fatigue, classified as a psychological symptom along with feeling low, irritability and feeling nervous, suggests it may be influenced by psychological factors, such as stress, anxiety, or emotional dysregulation^60,61^. Addressing mental health could thus be essential in managing fatigue, particularly in adolescents. Conversely, dizziness was the least prevalent symptom over the 20 years, although it reached its peak prevalence in 2022, with 10.3% of boys and 23.8% of girls reporting frequent dizziness. Sleeping difficulties showed the highest odds ratio, with Hungarian school-aged youth in 2022 being 161% more likely to experience frequent sleep difficulties than in 2002. This is line with the results published in the national Hungarian HBSC reports^62,63^, which point to a worsening trend between 2014 and 2018, followed by a dramatic decline in reported sleeping hours among pupils, from approximately 8 h (*SD* = 1.17 h) on school days in 2018 to 7.59 h (*SD* = 1.34 h) in 2022. Headache demonstrated the greatest gender difference, as girls were 245% more likely to experience frequent headaches compared to boys. From an international perspective^32^, headache and stomach-ache have shown remarkable increases among Hungarian adolescents. These findings underscore the critical need for targeted primary health interventions focusing on specific psychosomatic symptoms, such as headache and fatigue, in adolescent populations.

An increase in overall symptom load was also indicated. Our findings are in line with the results of other international studies^4,26,29^. The rising trend of MHC is crucial, as it indicates co-occurring and interrelated health issues. Compared to 2002, 2022 recorded the highest odds for MHC in both the 8- and 9-symptom criteria, with girls and older school-aged children reporting the highest odds. Analyses of the total scores on the HBSC-SCL provided additional insight: among boys, a significant increase began in 2014 for both the 8- and 9-symptom versions of the HBSC-SCL, with both reaching their highest levels in 2022. In contrast, among girls, a continuous upward trend started earlier—in 2010—for both versions, also culminating in peak scores in 2022. This trend underscores a steady rise in psychosomatic symptoms among Hungarian school-aged children, suggesting a potential deterioration in youth mental and physical health over time. Understanding this trend is crucial for identifying emerging health risks and developing targeted interventions to address factors such as stress, academic pressure, and lifestyle changes.

Investigating the explanatory variables contributing to the development of psychosomatic symptoms is beyond the scope of this study. Nonetheless, it is important to highlight some unique findings from psychological studies on Hungarian adolescents that may shed light on the worsening patterns of psychosomatic symptoms. The COVID-19 pandemic had a particularly profound impact on this population, with Hungarian adolescents reporting the second-highest prevalence (23%) of negative impacts and the third-lowest prevalence (13%) of positive impacts on various domains of life, including school performance and relationships with friends^64^. Unlike other countries, Hungary was the only nation among 22 surveyed where the positive effects of COVID-19 did not outweigh its negative effects in any domain of life. Bullying involvement has also been identified as a significant contributor to increased levels of psychosomatic symptoms, in both traditional and online forms^65^. Between 2002 and 2022, a notable—and in some years statistically significant—increase was observed in the proportion of Hungarian school-aged children who were regularly bullied, across both genders^63^. Additionally, while self-reported health status has declined—marked by the lowest proportion of adolescents rating their health as excellent in 2022 since 2002—the prevalence of chronic health conditions among pupils has remained consistent over this period^63^. It is important to note that the percentage of Hungarian school-aged children reporting a high level of pressure from schoolwork doubled between 2014 and 2022^63^, which may represent an important contributing factor. Descriptive analyses from the present study support this pattern: the proportion of pupils who perceived school tasks as highly pressuring increased steadily between 2002 and 2022, with a pronounced rise among girls after 2018. Although not analysed as a predictor, this trend mirrors the escalation in psychosomatic symptoms and highlights school-related stress as a contextual factor deserving further investigation. Social media use among youth represents another critical factor associated with elevated risks of psychosomatic symptoms. This risk is evident not only among individuals directly affected by problematic social media use but also among those in broader at-risk groups compared to non-risk users^66^. Notably, among girls, the proportion categorized as problematic social media users increased significantly by 5% between 2018 and 2022^63^. Overall, the decline in mental health indicators can be attributed not only to individual psychological factors but also to broader socio-psychological and ecological influences. Taken together, the observed increase in psychosomatic symptoms among Hungarian school-aged children highlights the importance of monitoring psychological and somatic symptoms, as well as the need for targeted mental health interventions. Gender- and age-specific approaches are essential, given the higher prevalence among girls and older adolescents. The sharp increase in reports of frequent low mood and irritability, especially among girls, warrants consideration, particularly in light of other studies indicating that the prevalence of elevated depressive symptoms (i.e. the risk of depression) increased from 24 to 37% in the 10–19 age group between 2001–2010 and 2011–2020^42^. Mobile apps aiming to reduce (psychological or depressive) symptoms show promise; however, evidence is currently inconclusive^67^. Based on the present findings screening for frequent fatigue, sleeping difficulties, and pain symptoms (headache, stomach-ache, backache) is also recommended, alongside addressing stress and emotional regulation, as subjective health complaints have been theorised to be at least partly related to psychosocial stress^68^. School-based programmes that promote stress management^69^ can reduce stress and increase resilience, albeit with a small effect size. However more studies needed to determine their broader impact on mental health (including symptoms or well-being).

### Major strengths, limitations, and future directions

A major strength of this study is that we used a large, nationally representative dataset collected over a 20-year period, and the application of multiple statistical methods (chi-square tests, logistic regression and ANCOVAs) produced converging results across analytical approaches, which increases confidence in the robustness of the patterns observed.

Despite the significant findings, this study is not without limitations. A widely recognized and valid self-report measurement (HBSC-SCL) to assess psychosomatic symptoms was used, which, as a subjective factor, cannot be captured using other methodologies. However, the reliance on self-reported data introduces the potential for bias, as children may underreport or overreport their symptoms due to factors such as social desirability or misinterpretations of their bodily or emotional experiences^70^. Additionally, the HBSC-SCL captures self-reported complaints rather than clinically verified conditions, and dichotomizing symptoms into frequent versus non-frequent may reduce information on severity gradients; these measurement features should be considered when interpreting the magnitude of change over time. It is important to note that the 2002 data were unweighted, yet this does not impact the fundamental nature of the trend when the 2002 data is excluded. In other years, minor discrepancies have been observed between weighted and unweighted data for these items. Given that our analysis is predominantly descriptive in nature, we contend that the absence of weighting in the 2002 data does not compromise the integrity of the overall results and trend. Furthermore, descriptive trend analyses, such as those conducted in the present study, are valuable as they offer insight into how symptom burden changes at the population level. As mentioned in the Methods section, the mode of administration in 2022 changed from paper-based to electronically administered, but a previous study demonstrated that it had no significant effect^48^ or only a minor significant effect in reporting exercise^49^ when comparing paper-based and electronically administered questionnaires among adolescents, suggesting that mode effects are likely small; however, we cannot fully exclude some influence of mode change on reporting. The cross-sectional design of the study provides a snapshot of data at one point in time. As the prevalence of frequent symptoms was examined, their relationships with other factors (e.g., academic stress, peer dynamics, family processes) that may have also varied during the investigated period (between 2002 and 2022) were not modelled statistically in this paper. In addition, given the large sample size, many comparisons reach statistical significance even when differences are modest; accordingly, statistical significance should be interpreted cautiously and with attention to the magnitude and precision of effects. In addition, Hungarian HBSC samples include 17-year-olds, unlike the international core HBSC target ages (11, 13 and 15), which should be considered when comparing the magnitude of temporal changes with international trend reports. Therefore, cross-national comparisons should be interpreted cautiously, particularly for late-adolescent patterns captured only in the Hungarian samples.

Finally, qualitative research examining the lived experiences of children reporting psychosomatic symptoms could offer valuable insights into the underlying factors that influence their health. A particularly valuable future design would be a cohort-sequential longitudinal approach that repeatedly assesses the same psychosocial (e.g,. school pressure) and contextual variables (e.g,. family structure) across successive birth cohorts, including personal-level characteristics such as emotion regulation strategies, in order to systematically identify which factors co-vary with symptom trajectories over time, and to determine whether these associations are reciprocal. Such designs would allow stronger tests of directionality and potential bidirectional pathways between stress-related exposures and symptom development.

## Conclusions

In conclusion, a worsening trend in psychosomatic symptoms has been observed between 2002 and 2022 among Hungarian school-aged children, particularly among girls and older children. The results suggest a marked increase in self-reported symptom burden among Hungarian youth. The findings highlight significant increases in symptoms such as feeling low, irritability, fatigue, sleeping difficulties, and headaches, with a notable peak in 2022, which coincides temporally with broader global concerns about the impact of the COVID-19 pandemic on youth mental health. These trends only partially align with international patterns, highlighting the need for country-specific analyses. The rising trend in MHC underscores the importance of strengthening prevention, screening, and support strategies addressing stress management and both mental and physical health for this vulnerable age group. Because the study is descriptive and repeated cross-sectional, the findings document trends but do not establish causes; future studies should investigate the underlying factors contributing to these concerning trends.

## Supplementary Information

Below is the link to the electronic supplementary material.

Supplementary Material 1

## Data Availability

The datasets generated and/or analysed during the current study are available in the Open Science Framework repository: 10.17605/OSF.IO/K2ASJ
