# Supplementary material for: Time trends in psychosomatic symptoms among Hungarian youth using repeated cross sectional HBSC data from 2002 to 2022
Source: Sci Rep. 2026 Feb 6;16:7569. doi: 10.1038/s41598-026-38472-0 (PMC12932817; doi:10.1038/s41598-026-38472-0)
Supplement: Supplementary file 1 — Supplementary Material 1 [file 41598_2026_38472_MOESM1_ESM.docx]

**Supplemental Materials**

**Time trends in psychosomatic symptoms among Hungarian youth using repeated cross sectional HBSC data from 2002 to 2022**

**Table S1** Pearson Correlation Between the Nine Psychosomatic Symptoms and Age

| Variables | 1 | 2 | 3 | 4 | 5 | 6 | 7 | 8 | 9 | 10 |
| --- | --- | --- | --- | --- | --- | --- | --- | --- | --- | --- |
| 1. Headache | 1 | .519** | .369** | .401** | .361** | .392** | .366** | .453** | .371** | .098** |
| 2. Stomach-ache |  | 1 | .387** | .402** | .382** | .391** | .376** | .446** | .359** | .058** |
| 3. Backache |  |  | 1 | .354** | .348** | .346** | .314** | .368** | .339** | .158** |
| 4. Feeling low |  |  |  | 1 | .611** | .598** | .445** | .398** | .489** | .148** |
| 5. Irritability |  |  |  |  | 1 | .680** | .400** | .373** | .443** | .183** |
| 6. Feeling nervous |  |  |  |  |  | 1 | .443** | .379** | .515** | .187** |
| 7. Sleeping difficulties |  |  |  |  |  |  | 1 | .421** | .413** | .082** |
| 8. Dizziness |  |  |  |  |  |  |  | 1 | .373** | .052** |
| 9. Fatigue |  |  |  |  |  |  |  |  | 1 | .179** |
| 10. Age |  |  |  |  |  |  |  |  |  | 1 |

*Note*. Total N = 37,930.

** p < .01.

**Table S2** Chi-Square Results for the Frequent Psychosomatic Symptoms Impacted by Survey Year, Separated by Gender

| Psychosomatic symptoms | Boys | |  | Girls | |
| --- | --- | --- | --- | --- | --- |
|  | N | χ2 (df = 5) |  | N | χ2 (df = 5) |
| Frequent headache | 18,305 | 101.82*** |  | 19,017 | 121.74*** |
| Frequent stomach-ache | 18,259 | 90.44*** |  | 18,970 | 226.97*** |
| Frequent backache | 18,185 | 113.77*** |  | 18,901 | 289.73*** |
| Frequent feeling low | 18,221 | 197.12*** |  | 18,937 | 630.57*** |
| Frequent irritability | 18,191 | 119.01*** |  | 18,942 | 625.48*** |
| Frequent feeling nervous | 18,231 | 197.39*** |  | 18,915 | 585.51*** |
| Frequent sleeping difficulties | 18,188 | 250.36*** |  | 18,921 | 589.96*** |
| Frequent dizziness | 18,125 | 55.87*** |  | 18,868 | 312.94*** |
| Frequent fatigue | 18,232 | 230.93*** |  | 18,929 | 691.38*** |

*Note*. A symptom was considered frequent when it was reported as occurring at least ‘More than once a week’ over the past six months.

*** p < .001.

**Table S3** Binary Logistic Regression Results of the Frequent Psychosomatic Symptoms by Gender, Survey Year and Age, With 95% Confidence Intervals

| Variables | Frequent  headache | Frequent  stomach-ache | Frequent  backache | Frequent  feeling low | Frequent  irritability | Frequent  feeling nervous | Frequent  sleeping difficulties | Frequent  dizziness | Frequent  fatigue |
| --- | --- | --- | --- | --- | --- | --- | --- | --- | --- |
|  | OR [95% CI] | OR [95% CI] | OR [95% CI] | OR [95% CI] | OR [95% CI] | OR [95% CI] | OR [95% CI] | OR [95% CI] | OR [95% CI] |
| **Gender** (ref = Boys) | |  |  |  |  |  |  |  |  |
| Girls | 3.45*** [2.95–4.02] | 2.91*** [2.42–3.48] | 1.65*** [1.40–1.94] | 2.04*** [1.79–2.32] | 1.48*** [1.30–1.70] | 1.46*** [1.30–1.63] | 1.70*** [1.46–1.98] | 2.39*** [1.95–2.93] | 1.61*** [1.44–1.80] |
| **Survey Year** (ref = 2002) | |  |  |  |  |  |  |  |  |
| 2006 | 1.66*** [1.40–1.96] | 1.34** [1.09–1.65] | 1.21* [1.02–1.45] | 1.25** [1.08–1.44] | 1.06 [0.91–1.22] | 1.06 [0.94–1.20] | 1.18* [1.00–1.39] | 1.33* [1.06–1.67] | 1.32*** [1.18–1.49] |
| 2010 | 1.68*** [1.43–1.96] | 1.59*** [1.32–1.92] | 1.33*** [1.14–1.57] | 1.01 [0.88–1.15] | 1.05 [0.92–1.20] | 0.96 [0.85–1.07] | 1.42*** [1.23–1.65] | 1.62*** [1.32–1.99] | 1.35*** [1.22–1.51] |
| 2014 | 1.35*** [1.14–1.61] | 1.34** [1.09–1.64] | 1.32** [1.11–1.57] | 1.15* [1.00–1.33] | 0.81** [0.70–0.94] | 0.77*** [0.68–0.87] | 1.32*** [1.13–1.55] | 1.35** [1.08–1.70] | 1.41*** [1.26–1.58] |
| 2018 | 1.56*** [1.31–1.85] | 1.58*** [1.29–1.92] | 1.44*** [1.21–1.70] | 1.28*** [1.11–1.47] | 1.08 [0.94–1.25] | 1.01 [0.89–1.14] | 1.73*** [1.48–2.02] | 1.46*** [1.17–1.83] | 1.70*** [1.52–1.91] |
| 2022 | 2.14*** [1.82–2.51] | 2.23*** [1.84–2.69] | 2.11*** [1.79–2.48] | 2.07*** [1.81–2.36] | 1.63*** [1.43–1.87] | 1.67*** [1.49–1.87] | 2.61*** [2.25–3.02] | 2.05*** [1.66–2.53] | 2.15*** [1.92–2.41] |
| **Age** | 1.07*** [1.06–1.08] | 1.03*** [1.02–1.04] | 1.11*** [1.10–1.13] | 1.10*** [1.09–1.11] | 1.13*** [1.12–1.14] | 1.13*** [1.12–1.15] | 1.05*** [1.04–1.06] | 1.03*** [1.02–1.05] | 1.14*** [1.13–1.15] |
| **Gender x Survey Year** (ref = Boys x 2002) | | |  |  |  |  |  |  |  |
| Girls x 2006 | 0.73** [0.60–0.90] | 0.92 [0.72–1.18] | 0.96 [0.76–1.20] | 0.80* [0.66–0.96] | 0.99 [0.82–1.21] | 0.98 [0.83–1.16] | 0.87 [0.71–1.08] | 0.75* [0.57–0.99] | 0.85 [0.73–1.00] |
| Girls x 2010 | 0.63*** [0.52–0.76] | 0.68*** [0.55–0.85] | 0.87 [0.71–1.07] | 0.73*** [0.62–0.87] | 0.85 [0.71–1.01] | 0.84* [0.72–0.98] | 0.75** [0.62–0.91] | 0.63*** [0.49–0.81] | 0.78*** [0.67–0.90] |
| Girls x 2014 | 0.81* [0.66–1.00] | 0.84 [0.66–1.07] | 1.02 [0.82–1.27] | 0.87 [0.73–1.05] | 1.41*** [1.17–1.71] | 1.17 [0.99–1.38] | 1.06 [0.87–1.30] | 0.69** [0.53–0.91] | 0.97 [0.83–1.13] |
| Girls x 2018 | 0.75** [0.62–0.92] | 0.85 [0.68–1.08] | 0.93 [0.75–1.16] | 0.98 [0.82–1.17] | 1.21* [1.01–1.46] | 1.17 [1.00–1.38] | 1.06 [0.87–1.29] | 0.87 [0.66–1.13] | 0.96 [0.82–1.12] |
| Girls x 2022 | 0.78* [0.64–0.94] | 0.95 [0.76–1.19] | 1.14 [0.93–1.40] | 1.17 [0.99–1.39] | 1.69*** [1.42–2.02] | 1.43*** [1.22–1.66] | 1.12 [0.93–1.35] | 1.14 [0.89–1.47] | 1.45*** [1.24–1.69] |

*Note*. A symptom was considered frequent when it was reported as occurring at least ‘More than once a week’ over the past six months. The reference categories are boys for gender, the year 2002 for the survey year, and boys x 2002 for the gender x survey year interaction. The variables were entered into the model using the Enter method. CI = Confidence Interval, LL = Lower Limit, UL = Upper Limit, OR = Odds Ratio.

* p < .05, ** p < .01, *** p < .001.

**S4** Percentages of Pupils Reporting the 8-Symptom Version of MHC (Without Fatigue) by Survey Year and Gender

|  | Boys | | Girls | |
| --- | --- | --- | --- | --- |
| Survey Year | % | N | % | N |
| 2002 | 25.7 | 697 | 41.6 | 1,338 |
| 2006 | 30.1 | 832 | 45.1 | 1,179 |
| 2010 | 30.2 | 1,245 | 40.6 | 1,584 |
| 2014 | 27.0 | 783 | 45.1 | 1,418 |
| 2018 | 31.2 | 907 | 48.7 | 1,470 |
| 2022 | 42.0 | 1,248 | 65.6 | 2,080 |

*Note.* Percentages represent the proportion; N indicates the number of boys and girls within each survey year. MHC = multiple health complaints, without fatigue.

**Table S5** Percentages of Boys Reporting the 8-Symptom Version of MHC (Without Fatigue) by Survey Year and Grades

|  | **5th** | | **7th** | | **9th** | | **11th** | |
| --- | --- | --- | --- | --- | --- | --- | --- | --- |
| **Survey Year** | **%** | **N** | **%** | **N** | **%** | **N** | **%** | **N** |
| 2002 | 23.2 | 157 | 27.2 | 191 | 27.8 | 151 | 25.1 | 198 |
| 2006 | 28.0 | 184 | 27.1 | 188 | 32.8 | 242 | 32.2 | 218 |
| 2010 | 27.7 | 242 | 26.5 | 249 | 33.1 | 380 | 32.2 | 374 |
| 2014 | 24.9 | 156 | 26.9 | 180 | 28.6 | 239 | 27.0 | 207 |
| 2018 | 29.9 | 215 | 26.4 | 171 | 33.8 | 267 | 33.9 | 253 |
| 2022 | 38.1 | 299 | 34.4 | 200 | 44.7 | 376 | 49.0 | 374 |

*Note.* Percentages represent the proportion; N indicates the number of boys within each survey year.

5th = 5th grade; 7th = 7th grade; 9th = 9th grade; 11th = 11th grade.

In the Hungarian HBSC, 17-year-olds (11th grade) are included; internationally, only 11-, 13-, and 15-year-olds (5th/7th/9th grades) are mandatory.

**Table S6** Percentages of Girls Reporting the 8-Symptom Version of MHC (Without Fatigue) by Survey Year and Grades

|  | **5th** | | **7th** | | **9th** | | **11th** | |
| --- | --- | --- | --- | --- | --- | --- | --- | --- |
| **Survey Year** | **%** | **N** | **%** | **N** | **%** | **N** | **%** | **N** |
| 2002 | 31.0 | 226 | 38.8 | 313 | 47.0 | 407 | 48.1 | 392 |
| 2006 | 32.7 | 192 | 44.3 | 262 | 49.9 | 383 | 51.1 | 342 |
| 2010 | 31.3 | 257 | 35.6 | 310 | 43.2 | 461 | 48.7 | 556 |
| 2014 | 29.7 | 191 | 45.5 | 290 | 50.6 | 472 | 49.9 | 464 |
| 2018 | 34.2 | 245 | 40.4 | 279 | 57.0 | 519 | 60.9 | 427 |
| 2022 | 42.7 | 306 | 66.7 | 369 | 72.3 | 825 | 76.4 | 580 |

*Note.* Percentages represent the proportion; N indicates the number of boys within each survey year.

5th = 5th grade; 7th = 7th grade; 9th = 9th grade; 11th = 11th grade.

In the Hungarian HBSC, 17-year-olds (11th grade) are included; internationally, only 11-, 13-, and 15-year-olds (5th/7th/9th grades) are mandatory.

**Fig. S1** Changes in the Percentage of Boys Reporting the 8-Symptom Version of MHC (Without Fatigue) over Time, Separated by Grades


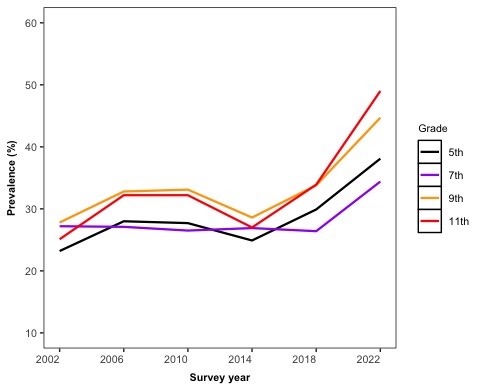


*Note*. MHC = multiple health complaints, including fatigue. 5th = 5th grade; 7th = 7th grade; 9th = 9th grade; 11th = 11th grade.

In the Hungarian HBSC, 17-year-olds (11th grade) are included; internationally, only 11-, 13-, and 15-year-olds (5th/7th/9th grades) are mandatory.

**Fig. S2** Changes in the Percentage of Girls Reporting the 8-Symptom Version of MHC (Without Fatigue) over Time, Separated by Grades


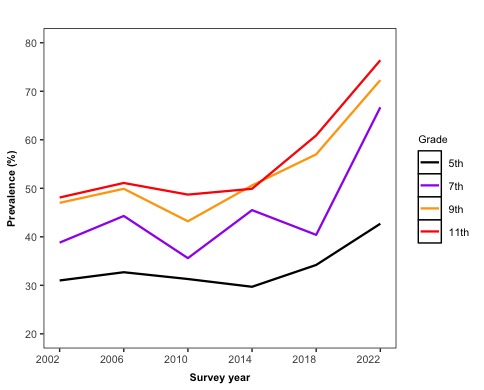


*Note.* MHC = multiple health complaints, including fatigue. 5 = 5th grade; 7th = 7th grade; 9th = 9th grade; 11th = 11th grade.

In the Hungarian HBSC, 17-year-olds (11th grade) are included; internationally, only 11-, 13-, and 15-year-olds (5th/7th/9th grades) are mandatory.

**Table S7** Percentages of Pupils Reporting the 9-Symptom Version of MHC (With Fatigue) by Survey Year and Gender

|  | Boys | | Girls | |
| --- | --- | --- | --- | --- |
| Survey Year | % | N | % | N |
| 2002 | 32.3 | 876 | 47.7 | 1,534 |
| 2006 | 36.4 | 1,008 | 52.1 | 1,363 |
| 2010 | 37.4 | 1,542 | 47.5 | 1,853 |
| 2014 | 34.7 | 1,005 | 52.5 | 1,652 |
| 2018 | 39.4 | 1,144 | 55.5 | 1,676 |
| 2022 | 50.0 | 1,484 | 71.7 | 2,274 |

*Note.* Percentages represent the proportion; N indicates the number of boys and girls within each survey year. MHC = multiple health complaints, with fatigue.

**Table S8** Results of Binary Logistic Regression for the 9-Symptom Version of MHC (With Fatigue)

| Variables | OR [95% CI] |
| --- | --- |
| **Gender** (ref = Boys) |  |
| Girls | 1.97*** [1.88, 2.05] |
| **Survey Year** (ref = 2002) |  |
| 2006 | 1.18*** [1.09, 1.28] |
| 2010 | 1.06 [0.99, 1.14] |
| 2014 | 1.10* [1.02, 1.18] |
| 2018 | 1.33*** [1.23, 1.43] |
| 2022 | 2.33*** [2.16, 2.51] |
| **Age** | 1.13*** [1.12, 1.14] |

*Note.* The reference categories are boys for gender and 2002 for the survey year. The variables were entered into the model using the Enter method. MHC = multiple health complaints, with fatigue. OR = Odds Ratio, CI = Confidence Interval.

* p < .05, *** p < .001.

**Fig. S3** Changes in the Percentage of Pupils Reporting the 9-Symptom Version of MHC (with Fatigue) over Time, Separated by Gender


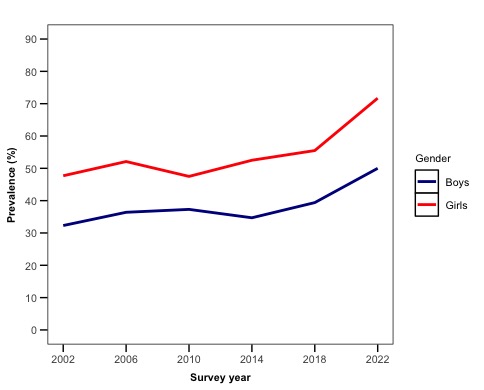


*Note*. MHC = multiple health complaints, including fatigue.

**Supplement S4. Total Scores for the 8- and 9-Symptom Versions of HBSC-SCL**

One-way ANCOVAs were performed to assess the impact of survey year on the total scores of the 8- and 9-symptom versions of the HBSC-SCL, with age serving as a covariate. Survey year was used as a polynomial variable to identify the trend patterns of the symptoms.

**Supplement S4.1 Total Scores for the 8-Symptom Versions of HBSC-SCL**

Descriptive statistics

Among boys the symptoms reached their lowest values in 2002 (*M* = 15.65, *SD* = 5.68) and their highest values in 2022 (*M* = 18.60, *SD* = 7.29), indicating a moderate increase between 2002 and 2022, with Cohen’s *d =* 0.45. In line with these results the symptoms reported by girls indicated their lowest levels in 2002 (*M* = 18.21, *SD* = 6.63) and their highest levels in 2022 (*M* = 23.09, *SD* = 8.02). A moderate increase of the symptoms was shown between 2002 and 2022, with Cohen’s *d =* 0.70. The descriptive statistics are presented in Table ~~S7~~S9.

**Table S9** Descriptive Statistics for the Total Score of the 8-Symptom Version of HBSC-SCL (Without Fatigue) by Survey Year and Gender

| Survey Year | Boys | | |  | Girls | | |
| --- | --- | --- | --- | --- | --- | --- | --- |
|  | M | SD | N |  | M | SD | N |
| 2002 | 15.65 | 5.68 | 2,468 |  | 18.21 | 6.63 | 3,011 |
| 2006 | 16.27 | 6.10 | 2,535 |  | 18.61 | 6.64 | 2,709 |
| 2010 | 16.50 | 6.43 | 4,279 |  | 18.17 | 6.78 | 3,900 |
| 2014 | 16.16 | 6.20 | 2,746 |  | 19.29 | 6.94 | 2,977 |
| 2018 | 16.88 | 6.76 | 2,827 |  | 19.80 | 7.39 | 2,971 |
| 2022 | 18.60 | 7.29 | 2,975 |  | 23.09 | 8.02 | 3,142 |

*Note*. HBSC-SCL = Health Behaviour in School-Aged Children – Symptom Checklist, without fatigue.

ANCOVA

**Boys:** The overall model was significant, *F*(6, 17,823) = 110.70, *p* < .001, and explained 3.6% of the variance in symptoms (*R²* = .036, adjusted *R²* = .036). The covariate age was significantly related to the symptom scores, *F*(1, 17,823) = 299.98, *p* < .001, partial *η*² = .017. After controlling for age, there was a statistically significant effect of survey year on symptoms, *F*(5, 17,823) = 71.42, *p* < .001, partial *η*² = .020, with a small effect size. The symptom trajectory was best characterized by a non-linear pattern, as revealed by significant linear, quadratic, cubic, and fifth-order trends (all *p* < .001). Bonferroni-adjusted post hoc comparisons reflected the following pattern: a significant increase in symptom scores between 2002 and 2006 (*p* = .014), followed by non-significant changes from 2006 to 2010 and from 2010 to 2014. From 2014 onward, symptom levels increased significantly, with marked rises observed between 2014 and 2018, and again between 2018 and 2022 (both *p* < .001). **Girls**: The overall model was significant, *F*(6, 18,703) = 384.35, *p* < .001, partial *η*² = .110. The model explained 11.0% of the variance in symptoms (*R²* = .110, Adjusted *R²* = .109). Age as covariate had a significant effect on the symptoms, *F*(1, 18,703) = 1144.66, *p* < .001, partial *η*² = .058. There was also a significant effect of survey year on symptoms after controlling for age, *F*(5, 18,703) = 220.41, *p* < .001, partial *η*² = .056, suggesting a moderate effect of the survey year. Again, changes in symptom levels between 2002 and 2022 were non-linear, as reflected in significant linear, quadratic, cubic, and fifth-order trends (all *p* < .001). Nevertheless, Bonferroni-adjusted post hoc tests revealed the following pattern: No significant difference in symptom scores was observed between 2002 and 2006; a significant decrease occurred from 2006 to 2010 (*p* = .011); this was followed by a continuous and significant increase from 2010 to 2022 - specifically between 2010 and 2014, 2014 and 2018, and 2018 and 2022, with all *p* < .001. Fig. ~~S2~~S4 illustrates the adjusted mean symptom levels for the six survey years for both genders, controlling for age.

**Fig. S4** The Age-Adjusted Mean Levels of the 8-Symptom Version of the HBSC-SCL (Without Fatigue), Separated by Gender


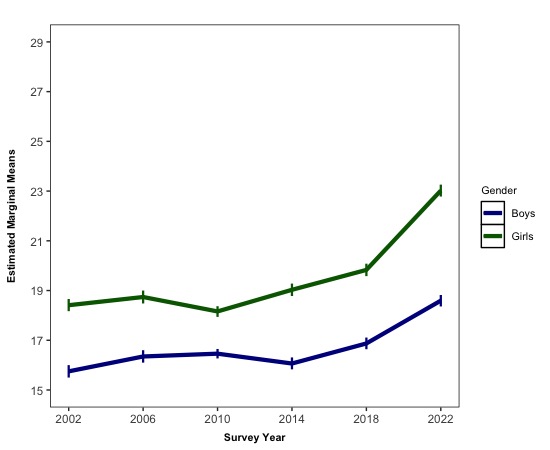


*Note*. Age is evaluated at the following values: age = 14.89 years. Fatigue excluded. The ticks represent 95% confidence intervals.

**Supplement S4.2 Total Scores for the 9-Symptom Version of HBSC-SCL**

Descriptive statistics

For boys, the symptoms reached their lowest values in 2002 (*M* = 18.30, *SD* = 6.45) and their highest values in 2022 (*M* = 21.85, *SD* = 8.19). There was a moderate increase of the symptoms between 2002 and 2022: Cohen’s *d* (2002 vs. 2022) = 0.48. Girls' reports showed the same pattern for the symptoms: the lowest values were reported in 2002 (*M* = 21.21, *SD* = 7.50), and the highest values in 2022 (*M* = 26.93, *SD* = 8.91). The increase between 2002 and 2022 was more pronounced among girls than among boys, but still moderate (Cohen’s *d* (2002 vs. 2022) = 0.69). The descriptive statistics are displayed in Table ~~S8~~S10.

**Table S10** Descriptive Statistics for the Total Score of the 9-Symptom Version of HBSC-SCL (With Fatigue) by Survey Year and Gender

|  | Boys | | |  | Girls | | |
| --- | --- | --- | --- | --- | --- | --- | --- |
| Survey Year | M | SD | N |  | M | SD | N |
| 2002 | 18.30 | 6.45 | 2,459 |  | 21.21 | 7.50 | 3,001 |
| 2006 | 19.14 | 6.92 | 2,533 |  | 21.71 | 7.53 | 2,708 |
| 2010 | 19.44 | 7.22 | 4,270 |  | 21.27 | 7.64 | 3,889 |
| 2014 | 19.15 | 7.03 | 2,745 |  | 22.61 | 7.82 | 2,973 |
| 2018 | 19.95 | 7.65 | 2,820 |  | 23.16 | 8.32 | 2,962 |
| 2022 | 21.85 | 8.19 | 2,973 |  | 26.93 | 8.91 | 3,138 |

*Note*. HBSC-SCL = Health Behaviour in School-Aged Children – Symptom Checklist, including fatigue.

ANCOVA

**Boys:** The overall model was statistically significant, *F*(6, 17,793) = 124.60, *p* < .001, partial *η*² = .040, and accounted for 4.0% of the variance in symptoms (*R²* = .040, Adjusted *R²* = .040). The covariate age had a significant effect on the symptoms, *F*(1, 17,793) = 350.99, *p* < .001, partial *η*² = .019. Survey year had a significant main effect on symptoms scores after controlling for age, *F*(5, 17,793) = 77.11, *p* < .001, partial *η*² = .021, reflecting a small effect size. The increase in symptoms across survey years was non-linear as evidenced by significant linear, quadratic, cubic, and fifth-order trends (*p* < .001). The Bonferroni-adjusted post hoc comparisons revealed a significant rise in symptom scores between 2002 and 2006 (*p* < .001), followed by non-significant changes between 2006 and 2010, as well as between 2010 and 2014. From 2014 onward, a significant upward trend emerged, with increases observed from 2014 to 2018 and from 2018 to 2022 (both *p* < .001). **Girls**: Again, the overall model was statistically significant, *F*(6, 18,664) = 419.50, *p* < .001, and explained 11.9% of the variance in symptoms (*R²* = .119, adjusted *R²* = .119). The covariate age was significantly related to the symptom scores, *F*(1, 18,664) = 1274.59, *p* < .001, partial *η*² = .064. Survey year had a significant effect on symptoms after controlling for age, *F*(5, 18,664) = 234.63, *p* < .001, partial *η*² = .059, however the effect size was moderate. The model suggested a non-liner trend, with *p* < .001 for linear, quadratic, cubic, and fifth-order trends. The Bonferroni multiple comparison method pointed out that no significant difference was observable between 2002 and 2006; but between 2006 and 2010 the symptoms decreased significantly (*p* = .023). From 2010 onwards, there was a continuous significant rise in the symptoms with p values (*p* < .001) for the periods 2010 to 2014, 2014 to 2018, and from 2018 and 2022. Fig. ~~S3~~S5 illustrates the adjusted mean symptom levels for the six survey years for both genders, controlling for age.

**Fig. S5** The Age-Adjusted Mean Levels of the 9-Symptom Version of the HBSC-SCL (With Fatigue), Separated by Gender


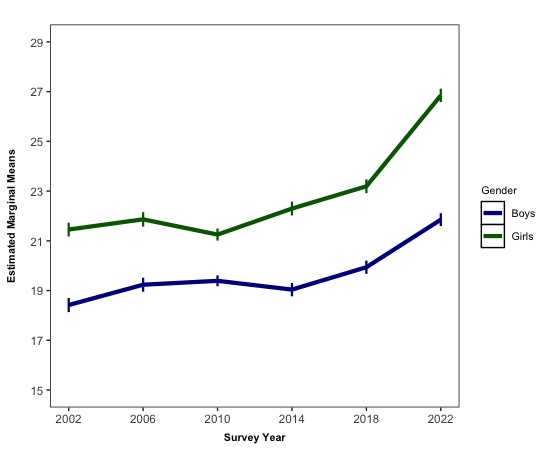


*Note*. Age is evaluated at the following values: age = 14.89 years. Fatigue including. The ticks represent 95% confidence intervals.

**Table** S11 Percentage and Frequency of Pupils Reporting High School Pressure by Gender and Survey Year

|  | Boys |  | Girls |  |
| --- | --- | --- | --- | --- |
| **Survey Year** | **%** | **N** | **%** | **N** |
| 2002 | 6.8 | 184 | 5.6 | 179 |
| 2006 | 8.0 | 206 | 5.7 | 162 |
| 2010 | 5.9 | 231 | 4.8 | 200 |
| 2014 | 5.2 | 157 | 5.1 | 157 |
| 2018 | 8.3 | 240 | 8.8 | 268 |
| 2022 | 9.5 | 291 | 14.1 | 451 |

*Note.* High school pressure reflects responses with a value of 4 on the school-pressure item.

**Fig. S6** Changes in the Percentage of Pupils Reporting High School Pressure over Time, Separated by Gender


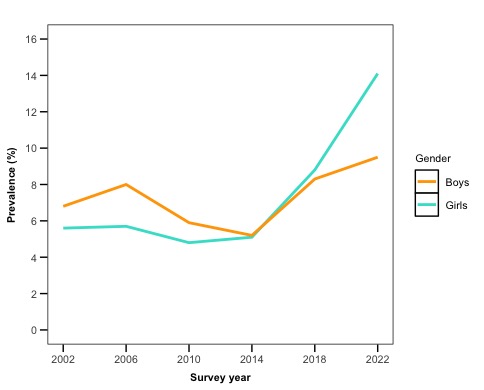


*Note.* High school pressure reflects responses with a value of 4 on the school-pressure item.
